# 1p36 deletion is a marker for tumour dissemination in microsatellite stable stage II-III colon cancer

**DOI:** 10.1186/1471-2407-14-872

**Published:** 2014-11-24

**Authors:** Markus Mayrhofer, Hanna Göransson Kultima, Helgi Birgisson, Magnus Sundström, Lucy Mathot, Karolina Edlund, Björn Viklund, Tobias Sjöblom, Johan Botling, Patrick Micke, Lars Påhlman, Bengt Glimelius, Anders Isaksson

**Affiliations:** Science for Life Laboratory, Department of Medical Sciences, Uppsala University, Box 3056, Uppsala, 750 03 Sweden; Department of Surgical Sciences, Uppsala University, Uppsala, Sweden; Science for Life Laboratory, Department of Immunology Genetics and Pathology, Uppsala University, Uppsala, Sweden; Department of Radiology, Oncology and Radiation Science, Uppsala University, Uppsala, Sweden; Leibniz Research Centre for Working Environment and Human Factors, Dortmund TU, Dortmund, Germany

**Keywords:** Colon cancer, Prognostic marker, Allele-specific copy number analysis, Genome duplication, 1p36, Metastasis, Tumour dissemination

## Abstract

**Background:**

The clinical behaviour of colon cancer is heterogeneous. Five-year overall survival is 50-65% with all stages included. Recurring somatic chromosomal alterations have been identified and some have shown potential as markers for dissemination of the tumour, which is responsible for most colon cancer deaths. We investigated 115 selected stage II-IV primary colon cancers for associations between chromosomal alterations and tumour dissemination.

**Methods:**

Follow-up was at least 5 years for stage II-III patients without distant recurrence. Affymetrix SNP 6.0 microarrays and allele-specific copy number analysis were used to identify chromosomal alterations. Fisher’s exact test was used to associate alterations with tumour dissemination, detected at diagnosis (stage IV) or later as recurrent disease (stage II-III).

**Results:**

Loss of 1p36.11-21 was associated with tumour dissemination in microsatellite stable tumours of stage II-IV (odds ratio = 5.5). It was enriched to a similar extent in tumours with distant recurrence within stage II and stage III subgroups, and may therefore be used as a prognostic marker at diagnosis. Loss of 1p36.11-21 relative to average copy number of the genome showed similar prognostic value compared to absolute loss of copies. Therefore, the use of relative loss as a prognostic marker would benefit more patients by applying also to hyperploid cancer genomes. The association with tumour dissemination was supported by independent data from the The Cancer Genome Atlas.

**Conclusion:**

Deletions on 1p36 may be used to guide adjuvant treatment decisions in microsatellite stable colon cancer of stages II and III.

**Electronic supplementary material:**

The online version of this article (doi:10.1186/1471-2407-14-872) contains supplementary material, which is available to authorized users.

## Background

Colon cancer is a heterogeneous disease in terms of clinical behaviour with an overall 5-year survival of 50-65%. Except for postoperative mortality all colon cancer-related deaths are caused by dissemination of the tumour (metastatic disease), present in 20-25% of patients at the time of diagnosis, and appearing to a similar extent during follow-up in individuals who were found to be metastasis-free at diagnosis. After surgical resection of the primary tumour, adjuvant chemotherapy may reduce the risk of subsequent relapse by eradicating subclinical tumour deposits. Prognostic markers are warranted in patient subgroups where they could influence the choice of treatment, such as selecting adjuvant therapy in stage II-III patients. TNM staging has relatively low predictive value, but is currently the only validated prognostic tool. Improved molecular prognostic markers could have a potential to reduce both over- and under-treatment by identifying patients with the greatest potential benefit from adjuvant therapy.

The mutational landscape of colon cancer has been explored in detail [1]. Colon cancers with microsatellite instability (MSI) have few or no somatic copy number alterations (CNAs). Microsatellite stable (MSS) colon cancers frequently have mutations in tumour suppressor genes such as APC and TP53. MSS colon cancers also frequently have chromosomal instability (CIN) which results in numerous CNAs. Multiple molecular prognostic markers such as MSI (excluding low-level MSI [2, 3]), loss of 18q and reduced SMAD4 expression have been suggested [4–7]. Other CNAs that have been associated with survival or tumour dissemination include loss of 1p, 4p, 8p, 9q, 10p 15q, 19p and 20p and gain of 8q and 20q [1, 8–10]. Unfortunately, findings vary considerably between studies and there is no consensus set of CNAs associated with tumour dissemination, i.e. prognosis for patients without metastasis at diagnosis.

Copy number analysis of tumour tissue is complicated by unknown ploidy of the tumour cells, by normal cells in the tumour tissue, and by subclonal CNAs. Bioinformatic tools such as TAPS [11] use bi-allelic probe signals from SNP arrays to estimate absolute allele-specific copy numbers in tumour cells. Allele-specific copy number analysis has been used to estimate frequency of hyperploidy and whole-genome duplication in multiple cancer types [12].

This study aimed to identify CNAs in colon cancer that may be used at diagnosis to predict risk for tumour dissemination in stage II-III patients. DNA from resected stage II-IV colon cancer primary tumours were analysed on Affymetrix SNP 6.0 arrays. Bioinformatic analysis identified deletion on 1p36 as a marker for tumour dissemination.

## Methods

### Study population

The study cohort included 116 patients operated for colorectal cancer between 1985 and 2006 at the Uppsala University hospital and at Västerås district general hospital between 2000-2003, with fresh frozen tissue samples available. We aimed at selecting between 20-25 cases each with stages II and III with and without distant recurrence and stage IV. Morphological and clinical parameters were retrieved from the original pathology reports. Patients with a history of preoperative therapy or with a surgical or pathology report suggesting a non-radical resection margin (R1 or R2 resection) were excluded. To secure the quality of disease staging, patients with stage II disease were only included if at least 10 lymph nodes were analysed. Patients with disease stage II-III and no recurrence were only included if the follow-up time was longer than 5 years. Tumour cell content was required to be at least 40% in the frozen tissue block. The study design was chosen to have few factors confounding an association between the tumour genome at diagnosis (surgery) and development of distant metastasis. Clinical and histological characteristics are presented in Table 1. Adjuvant chemotherapy, chiefly with a fluoropyrimidine alone was given to 22 out of 53 stage II-III patients without recurrence and to 29 out of 40 patients in stages II-III who developed distant metastasis.

**Table 1:** **Clinical and histopathological data**

	Total	Stage II-III	Stage II-IV	p
		No recurrence	Disseminated	
**Gender**				
Male	48	24	24	0.434
Female	68	29	39	
**Location**				
Right colon	70	33	37	0.698
Left colon	46	20	26	
**Differentiation**				
Well- Moderately	89	41	48	0.882
Poor	27	12	15	
**Stage**				
II	40	25	15	
III	53	28	25	
IV	23	-	23	
**Tumour size**				
<5 cm	37	12	25	0.058
≥5 cm	78	40	38	
Unknown	1	1		
**Mucinous**				
Yes	19	11	8	0.243
No	97	42	55	
**Perineural invasion**				
Yes	3	0	3	0.157*
No	113	53	60	
**Vascular invasion**				
Yes	16	6	10	0.479
No	100	47	53	
**Microsatellite instability**				
MSI-High	24	17	7	0.008
MSS or MSI-Low	92	35	57	

*Fisher’s exact test, otherwise χ^2^.

### DNA extraction

Genomic DNA was extracted from 10 μm sections of the fresh frozen tissue using QIAamp DNA mini kit (QIAGEN GmbH, Hilden, Germany) according to the manufacturer’s recommendations for DNA purification from tissue. Alternatively, genomic DNA was extracted from approximately 25 mg of each fresh frozen colon tissue sample on a Tecan Evo MCA 150 robotic platform using the extraction method described in Mathot *et al* [13]. DNA concentration was determined using NanoDrop (Thermo Scientific, Wilmington, DE).

### MSI analysis

MSI status was determined using MSI Analysis System, version 1.2 (Promega, Madison, WI) with 6 ng genomic DNA and analysis of five mononucleotide repeat markers (BAT25, BAT26, NR-21, NR-24 and MONO-27). Analyses were performed on a 3130xl genetic analyser (Applied Biosystems, Foster city, CA). According to guidelines from a National Cancer Institute workshop in 1997, samples were denoted MSI-High (MSI-H) if two or more of the five markers show instability, MSI-Low (MSI-L) if only one marker shows instability and microsatellite stable (MSS) if no markers display instability. Recent studies indicate no significant difference between MSI-L and MSS [14] and they were therefore grouped together as MSS in this study.

### Microarray analysis

Array experiments were performed according to standard protocols for Affymetrix GeneChip® Mapping SNP 6.0 arrays (Affymetrix Cytogenetics Copy Number Assay User Guide (P/N 702607 Rev2.), Affymetrix Inc., Santa Clara, CA). 500 ng total genomic DNA was digested with a restriction enzyme (Nsp, Sty), ligated to an appropriate adapter for the enzyme, and subjected to PCR amplification using a single primer. After digestion with DNase I, PCR products were labelled with a biotinylated nucleotide analogue using terminal deoxynucleotidyl transferase and hybridized to the microarray. Hybridized probes were captured by streptavidin-phycoerythrin conjugates using Fluidics Station 450 and arrays were scanned using GeneChip® Scanner 3000 7G. SNP array data generated in this study have been deposited at GEO with accession number GSE62875. Independent SNP 6.0 data from TCGA colon adenocarcinoma were retrieved from http://cancergenome.nih.gov.

### Data analysis and statistics

Basic normalisation and segmentation of the microarray data were performed using BioDiscovery Nexus Copy Number 6.0 and the SNP Rank Segmentation algorithm based on Circular Binary Segmentation [15] and default settings. Analyses of absolute allele-specific copy numbers, average ploidy and normal cell content were performed using TAPS [11]. Copy number estimates are included in the Additional file 1.

CNA frequencies (gain to >2 copies per cell, loss to <2 copies per cell, relative gain to >1.25* individual sample average copy number, relative loss to <0.67* individual sample average copy number, homozygous loss, high gain to >3 copies above individual sample average copy number, focal gain and loss of <1 Mb segments, and loss of heterozygosity) and group comparisons were generated using TAPS. Fisher’s exact test was used to estimate statistical significance of observations, generating unadjusted p-values and odds ratios for short segments throughout the genome such that none contained a breakpoint in any sample. A p-value of 0.05 was used as an initial cut-off for significance.

Average copy number or ploidy was calculated as the mean total copy number throughout the autosomes. The difference between the number of autosome arms with 2m0 (two copies with minor allele copy 0, i.e. LOH) or 4m2, and 2m1 or 4m1 was used as a score for evidence of a genome duplication event (using medians of total and minor allele copies throughout each autosome arm, Formula 1).
1

## Results

Copy number analysis was successful for 115 samples, of which 23 were MSI and 92 MSS. Tumour dissemination was defined as either stage IV with distant metastasis at diagnosis, or stage II-III and recurrence with distant metastasis within 5-years of diagnosis. No association was found between tumour dissemination and gender, tumour location, differentiation, tumour size, mucinous appearance, or neural or vascular invasion (Table 1).

**Figure 1:**
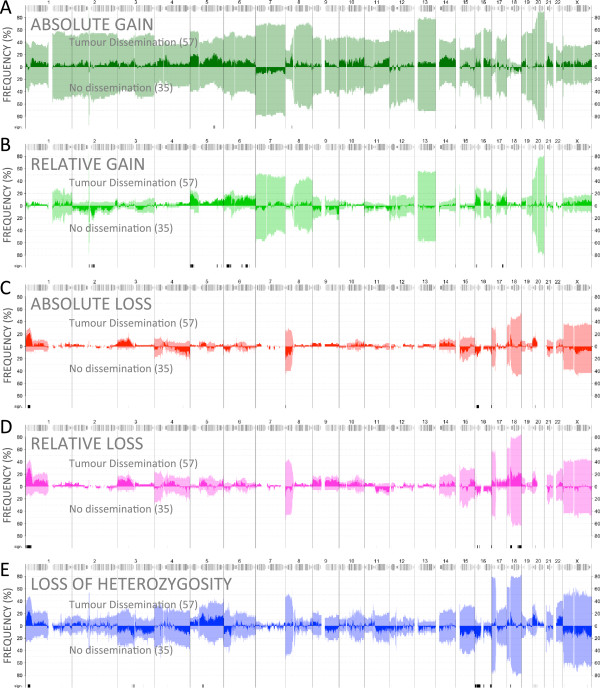
**Frequency of copy number alterations.** Microsatellite stable tumours with dissemination (stage II-III with distant recurrence, stage IV) and without (stage II-III no recurrence). Frequency difference is shown with a darker colour. Different types of CNAs were analysed separately: **A)** Gain (to >2 copies). **B)** Relative gain (to >25% above individual sample average copy number). **C)** Loss (to <2 copies). **D)** Relative loss (to <67% of individual sample average). **E)** Loss-of-heterozygosity (no minor allele copy). Regions with significant difference in alteration frequency (p < 0.05, Fisher’s exact test) are marked by black bars below each panel.

DNA from all samples was analysed using Affymetrix SNP 6.0 arrays and absolute allele-specific copy numbers in the cancer cells were estimated using TAPS [11]. All samples with MSI (n = 23) were near diploid with relatively few CNAs; median 3 chromosomes affected and median 87 Mb altered. Nearly all samples with MSS had an abundance of CNAs affecting large proportions of the genome; median 17 chromosomes affected and median 940 Mb altered. Hyperploidy, defined as an average copy number above 2.5, was observed in many MSS samples but showed no association with tumour dissemination (p = 0.33). Associations between specific CNAs and tumour dissemination were investigated separately in MSI and MSS due to the different frequencies of alterations. Sample groups with and without dissemination were compared for differences in the frequency of various types of CNAs (see Methods). Fisher’s exact test was used to generate p-values for the null hypothesis that the observed difference in CNA frequency is random. Alteration frequencies and frequency differences in MSS samples are shown in Figure 1.

In MSS samples, frequencies of CNAs were similar in samples with and without dissemination, with amplification being more frequent than deletion throughout most chromosomes (Figure 1A, C). Deletion was more frequent than amplification only on chromosomes 8p, 17p and 18. Frequencies of absolute loss (1C) resembled those of loss relative to the average copy number of the individual genome (1D) and LOH (1E) due to their natural overlap; e.g. absolute loss is also considered LOH. Regions with a significantly different frequency of alteration (p < 0.05, Fisher’s Exact Test) between the prognostic groups included 1p36 and 18q (relative loss), 2q12-14, 5p, 6pq and 17q (relative gain) and 16p (LOH). Additional file 1 includes a complete list of CNA frequency differences (including focal alterations and homozygous loss for which no potential markers were found) and corresponding p-values and odds ratios. In tumours with MSI, some genomic regions including 18q were more commonly altered in disseminated tumours, but the number of MSI samples was limited and statistical significance was not reached for any specific CNA (Additional file 1: Figure S1).

### 1p36 deletion is a marker for tumour dissemination in stages II and III

We used a publicly available set of colon adenocarcinoma samples from The Cancer Genome Atlas (TCGA) [1] to verify which of our findings could be observed in independent data. The TCGA data set differed from the current study in that progression after diagnosis and MSI status was documented only for subsets of the patients. We observed that large CNAs (≥10 Mb) were rare in MSI samples in the current study, affecting less than 5 chromosomes in all but one case, while >93% of MSS cases had CNAs on at least 5 chromosomes (Additional file 1: Figure S2A). TCGA samples with known MSI status showed a similar distribution (Additional file 1: Figure S2B). TCGA samples with unknown MSI status and at least 5 chromosomes affected (n = 37) were considered CIN and included with known MSS samples in order to maximise the number of samples available for validation. MSS/CIN TCGA cases with metastasis at diagnosis (n = 39) were compared with cases without metastasis at diagnosis and with documented survival greater than two years (2-10 years, mean = 5, n = 28). Out of all genomic regions with significantly different frequency of alteration and good effect size (odds ratio ≥4) in our data, only deletion on 1p36 (odds ratio ≈ 6) was independently significant in TCGA. We also observed that deletion or LOH on 18q11.2 (short segment including *CABLES1*, odds ratio ≈ 3) was associated with dissemination in both data sets, but with a relatively low effect size (Additional file 1).

**Figure 2:**
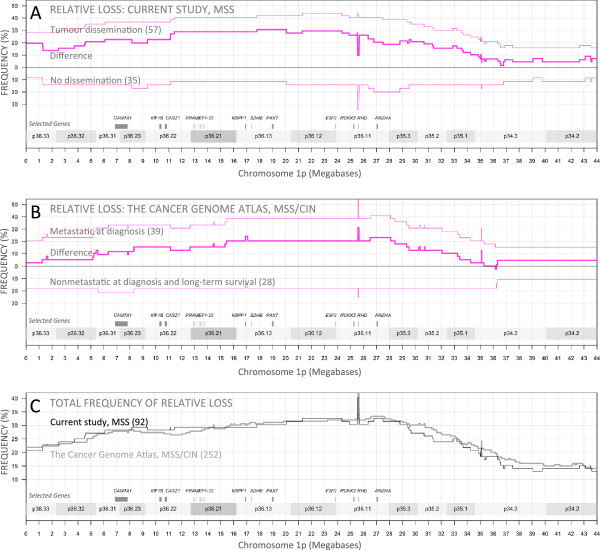
**Relative loss on 1p36. A)** Frequency of relative loss (<67% of individual sample average copy number) on 1p36 when comparing disseminated (metastatic SII-IV) and non-disseminated (SII-III) colon cancer, MSI excluded. High difference in frequency extended through 1p36.11-21 and included multiple cancer-related genes. **B)** TCGA validation set. Frequency of relative loss on 1p36, comparing stage IV cases with cases non-metastatic at diagnosis and with long-term survival. **C)** Total frequencies of relative loss were very similar in the current study and the TCGA validation set. Focal deletion of *RHD* (1p36.11) is a common germline polymorphism, likely not associated with the cancer.

Subsets (stage II and stage III separately and together, and with postoperative chemotherapy treated cases removed) of the current study displayed associations (odds ratios) very similar to those of stages II-IV combined, supporting that the deletion may be used as a prognostic marker at diagnosis. Peak odds ratios and independent 95% confidence intervals are shown in Table 2. Statistics at base-pair resolution throughout the region are included in Additional file 1. Absolute loss (fewer than 2 copies remaining) and relative loss (fewer than genome average number of copies remaining) were similarly associated with tumour dissemination, though relative loss was more frequently observed (Table 2). For relative loss on 1p36, the difference in frequency between the prognostic groups of the current study (stage II-IV) exceeded 30% on 1p36.13 but was similar throughout 1p36.11-21 (Figure 2A). For the TCGA validation set the difference in frequency exceeded 20% on 1p36.11 and was similar throughout 1p36.11-13 (2B). Total frequency of relative loss displayed very similar profiles in both data sets and peak frequency of loss could be observed near 27 Mb (2C). The difference in frequency between the prognostic groups could be confirmed in the TCGA data set (2B), but a single gene or region responsible for the worse prognosis could not be pinpointed.

**Table 2:** **Effect size of 1p36 loss association with tumour dissemination**

Alteration 1p36.11-21	Absolute loss	Relative loss
OR stage II-IV	4.5 (1.1-25.9)	5.5 (1.6-24.5)
OR stage II	4.4 (0.3-262)	6.5 (0.5-368)
OR stage III	3.6 (0.6-40.3)	3.3 (0.7-22.5)
OR stage II-III	4.0 (0.9-25.1)	4.3 (1.13-20.5)
OR stage II-III _no chemotherapy_	8.0 (0.9-393)	9.7 (1.1-472)
OR TCGA	4.1 (1.0-25.1)	2.8 (0.8-11.6)
Total frequency	17%	30%

### Duplication of the genome is not associated with prognosis or relative loss on 1p36

**Figure 3:**
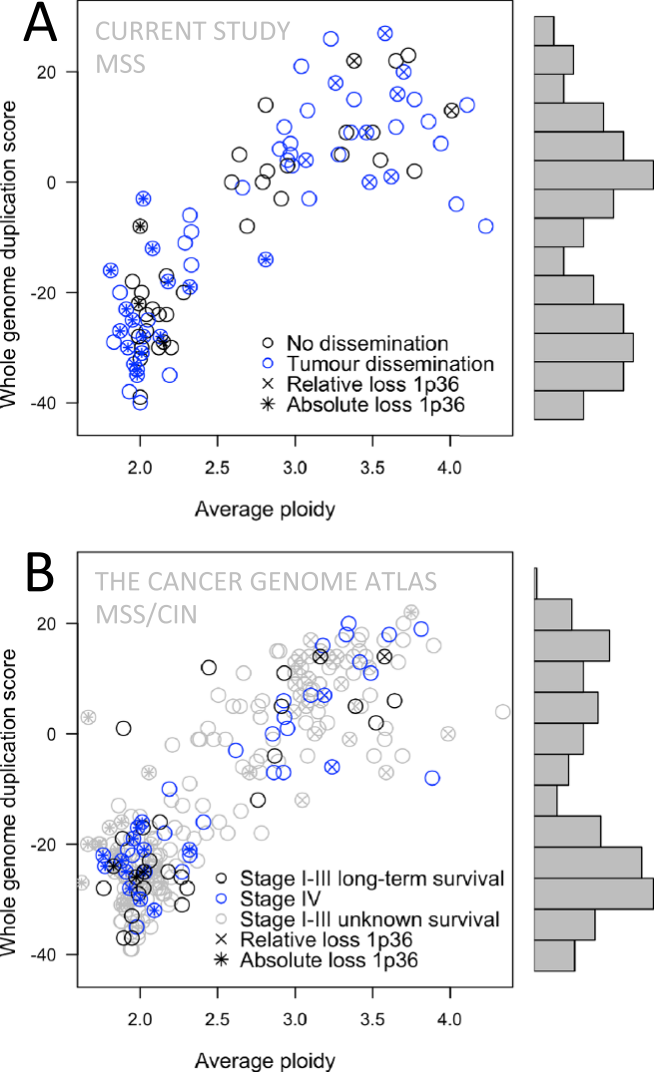
**Whole genome duplication.** Scatter plots of whole genome duplication (WGD) score and average ploidy indicate strong correlation between hyperploidy and evidence of genome duplication, neither of which associated with tumour dissemination or relative loss of 1p36. Absolute loss of 1p36 (which is also counted as relative loss) was almost exclusive to near-diploid genomes. The histograms show bi-modal distributions for the WGD score, which suggests two groups of samples; one having undergone WGD and the other not. **A)** Samples of the current study (MSS, 92) with tumour dissemination (metastatic disease) in blue. **B)** TCGA validation samples (MSS/CIN, 252), metastatic at diagnosis in blue and with no metastasis at diagnosis and long-term survival in black.

In both the current study and the TCGA validation data, 41% of MSS/CIN samples were found to have an average copy number or ploidy above 2.5. Hyperploid genomes may be the effect of whole genome duplications (WGD), which have been implicated in tumours and observed in cancer cell lines [16, 17]. Allele-specific copy number analysis has been used to identify WGD as a frequent event in a variety of cancers including colon cancer, where the frequency of WGD has been estimated to approximately 50% [12]. We investigated evidence of hyperploidy (average copy number >2.5) and WGD in the current study and in the TCGA validation set. For chromosomes present in 4 copies, a WGD event is more likely to have produced 2 copies of each homolog than triplication of one homolog, which is the more likely outcome of successive amplification events leading to 4 copies. Similarly where 2 copies are present, LOH is more likely observed if a WGD event has taken place (after loss of one copy, or followed by loss of two random copies) than if not. We used these assumptions to develop a score sensitive to WGD (see Methods) that would not be directly influenced by the average copy number of the genome. Not surprisingly, average ploidy correlated strongly with WGD score (Figure 3AB). Bimodal distributions of the WGD score for both the current study and the TCGA validation set suggest two groups of samples; one having undergone WGD and the other not. WGD appeared to have occurred in most genomes with hyperploidy and in about one third of all samples. Average ploidy or WGD score did not associate with prognosis or relative loss on 1p36 (p > 0.2, current study MSS, logistic regression). However, absolute loss on 1p36 was strongly associated with, and nearly exclusive to near diploid genomes (p < 10^-5^).

## Discussion

Treatment decisions for colon cancer patients are based on TNM staging, where stage III patients most often receive adjuvant chemotherapy while stage II patients (due to the low risk for metastatic relapse) are only treated beyond surgical resection if some risk factors are observed. Molecular markers have the potential to guide the use of adjuvant treatment to minimize over- and under-treatment.

Association between 1p36 loss and metastasis in colon cancer has been described previously [1, 18–20]. In this study we have shown that loss on 1p36 is associated with tumour dissemination in MSS tumours of stages II-IV. Stage IV was included in the study as cases with tumour dissemination in order to improve the total number of samples and the estimate of effect size, but significance was retained in the stage II-III subset. Statistical significance was also retained for stage II-III when patients who received adjuvant chemotherapy were excluded, and odds ratios were similar in stage II and stage III separately, supporting that 1p36 loss can be used as a prognostic marker at diagnosis. While it is not unlikely that this marker can be applied also to MSI cases (disseminated MSI cases were indeed enriched for CNAs similar to those seen in MSS, Additional file 1: Figure S1), the current study included too few MSI cases with dissemination to explore this further.

1p36.11-12 was the most commonly deleted region of 1p36 in both the current study and in the TCGA validation set. The strongest association with tumour dissemination was seen in a 15 Mbp region of 1p36.11-21 (Figure 2A), similar to the region identified by Thorstensen et al [19]. The region contains multiple genes with a known or suspected role in cancer including *ARID1A* [21], *E2F2* [22], *NBPF1* [23], *PAX7* [24], RUNX3 [25] and *SDHB* [26]. One or more such gene may be the cause of worse prognosis through a dosage effect. It should be possible to identify them using a larger number of samples, or using expression analysis of samples without loss of copies, as such a gene may also be down-regulated by other genetic or epigenetic mechanisms.

Assessing the practical value of a marker requires estimating the associated relative and absolute risk. In a selected case-control study such as this one only odds ratio is relevant, as the absolute frequency of distant recurrence and related markers cannot be estimated without an unselected cohort design.

Analysis of absolute allele-specific copy numbers is uncommon in studies of this kind; copy number gain and loss are normally assigned based on log-ratio only (DNA abundance in the extract, along the reference genome, relative to its own median [11]), disregarding tumour cell content and unknown average ploidy of genomes. Absolute loss of copies results in a relatively low copy number relative to the rest of the genome, but LOH or relative loss may occur without absolute loss to fewer than two copies. We identified relative loss on 1p36 as a better marker than absolute loss due to a combination of high odds ratio and high total frequency. Relative loss on 1p36 was not associated with hyperploidy or duplication of the genome, while absolute loss was almost exclusive to near diploid genomes. Though the prognostic values of absolute and relative loss on 1p36 were similar (Table 2), relative loss as a prognostic marker would benefit more patients by applying also to hyperploid cancer genomes. It should be noted that if a gene dosage effect is causing the worse prognosis, the effect on prognosis may depend on the size of the dosage effect (e.g. in a genome with four copies on average, 3 remaining copies of 1p36 may lead to a better prognosis than one remaining copy). A much larger number of samples would be required to describe such an effect in detail.

This study was designed to investigate association between genomic aberrations and tumour dissemination as a categorical variable, at diagnosis or within 5 years of observation after surgery, and irrespective of time to recurrence or death. Five years is a sufficiently long time to identify virtually all recurrences in colon cancer patients [27]. Relative loss on 1p36 may be a particularly useful prognostic marker for stage II patients where it, according to our results, motivates the use of adjuvant chemotherapy and regular observation for signs of relapse.

## Conclusions

In this study we have shown that 1p36 deletion can be used to predict metastatic recurrence in stage II-III patients. The association with metastatic disease was validated in independent data from The Cancer Genome Atlas. Allele-specific copy number analysis allowed the distinction of 1p36 loss relative to individual genome average ploidy as a better prognostic marker than absolute loss of copies, as relative loss had similar prognostic value and was more frequent. This marker may be used to reduce under-treatment particularly in stage II where about 15% of patients have distant recurrence after treatment primarily based on surgery.

## Ethical approval

This study was approved by the Regional Ethical Review Board of Uppsala (2007/116) and written consent was obtained from participants.

## Electronic supplementary material

Additional file 1: **This file includes supplementary figures, genome-wide copy number estimates and statistics.** (ZIP 5 MB)

## Abbreviations

CIN: Chromosome Instability
CNA: Copy Number Alteration
LOH: Loss-of-heterozygosity
MSI: Microsatellite Instability
MSS: Microsatellite Stable
OR: Odds Ratio
TCGA: The Cancer Genome Atlas
WGD: Whole Genome Duplication.
