# Supplementary material for: 1p36 deletion is a marker for tumour dissemination in microsatellite stable stage II-III colon cancer
Source: BMC Cancer. 2014 Nov 24;14:872. doi: 10.1186/1471-2407-14-872 (PMC4251789; doi:10.1186/1471-2407-14-872)
Supplement: Supplementary file 1 — Additional file 1: This file includes supplementary figures, genome-wide copy number estimates and statistics. (ZIP 5 MB) [file 12885_2014_5046_MOESM1_ESM.zip › Supplementary Data/Supplementary Figure Legends.docx]

# Supplementary Figure Legends

Supplemetary Figure S1. Frequency of copy number alterations (CNAs) in patients with tumour dissemination (stage II-III with distant recurrence, stage IV) and without (stage II-III no recurrence) in microsatellite instability (MSI) samples. Frequency difference is shown with a darker colour. Different types of CNAs were analysed separately: A) Gain (to >2 copies). B) Relative gain (to >25% above individual sample average copy number). C) Loss (to <2 copies). D) Relative loss (to <67% of individual sample average). E) Loss-of-heterozygosity (no minor allele copy). Regions with significant difference in alteration frequency (p<0.05, Fisher’s exact test) are marked by black bars below each panel.

Supplemetary Figure S2. Number of autosomes with large copy number alterations (>10 Mbp) in colon cancers with microsatellite instability (MSI) and microsatellite stability (MSS). TCGA samples with less than five chromosomes affected and unknown MSI status were excluded from the validation of prognostic markers in MSS colon cancer.

Supplemetary Figure S3. Frequency of copy number alterations (CNAs) in TCGA samples with tumour dissemination at diagnosis (stage IV) and without (and long-term survival), using microsatellite stable and CIN samples. Frequency difference is shown with a darker colour. Different types of CNAs were analysed separately: A) Gain (to >2 copies). B) Relative gain (to >25% above individual sample average copy number). C) Loss (to <2 copies). D) Relative loss (to <67% of individual sample average). E) Loss-of-heterozygosity (no minor allele copy). Regions with significant difference in alteration frequency (p<0.05, Fisher’s exact test) are marked by black bars below each panel.
