# Supplementary figures and images for: 1p36 deletion is a marker for tumour dissemination in microsatellite stable stage II-III colon cancer
Source: BMC Cancer. 2014 Nov 24;14:872. doi: 10.1186/1471-2407-14-872 (PMC4251789; doi:10.1186/1471-2407-14-872)

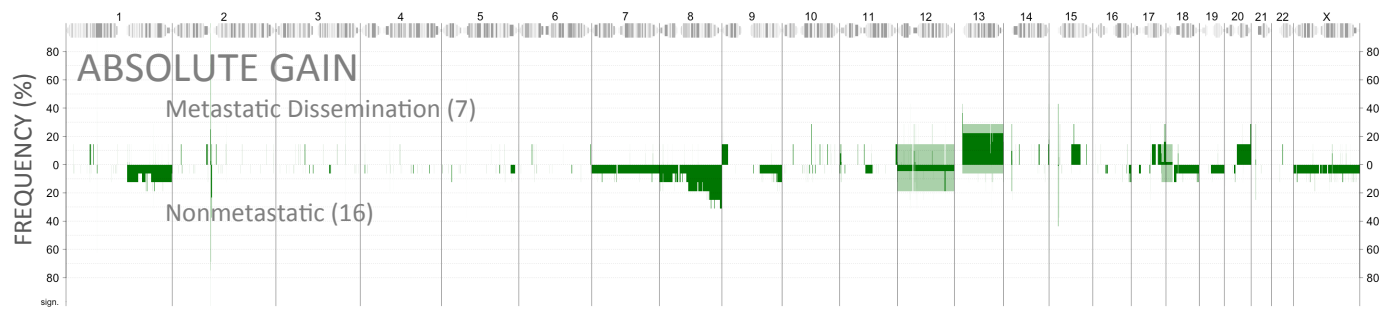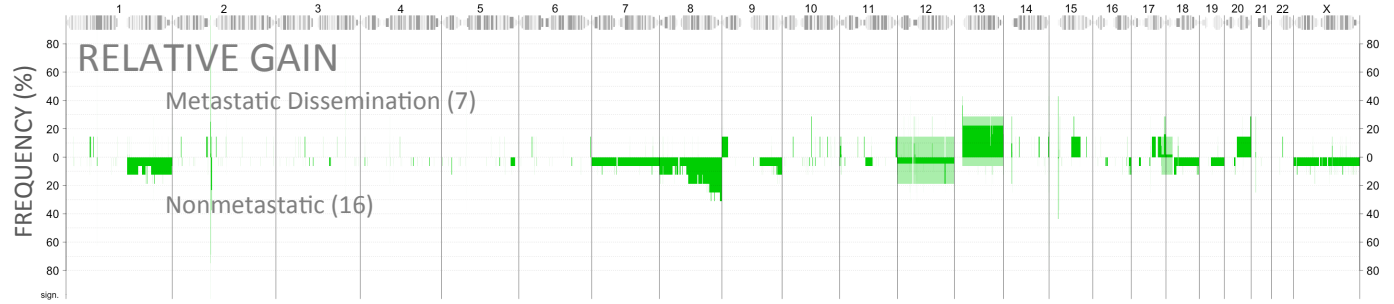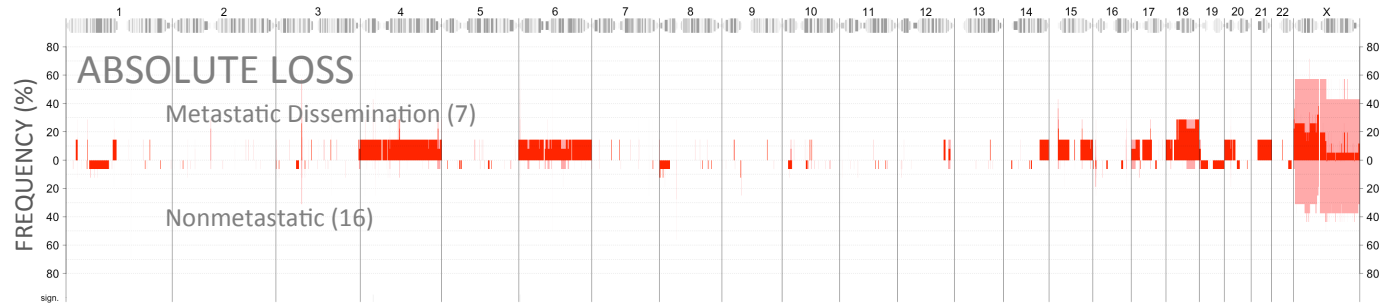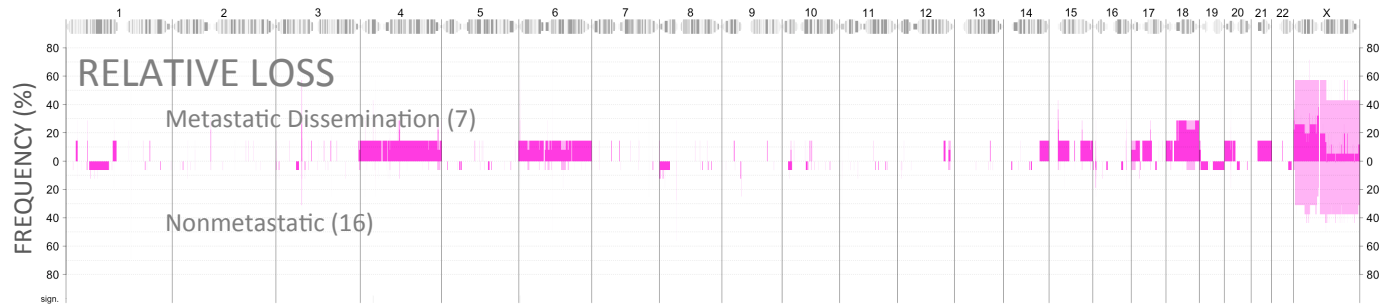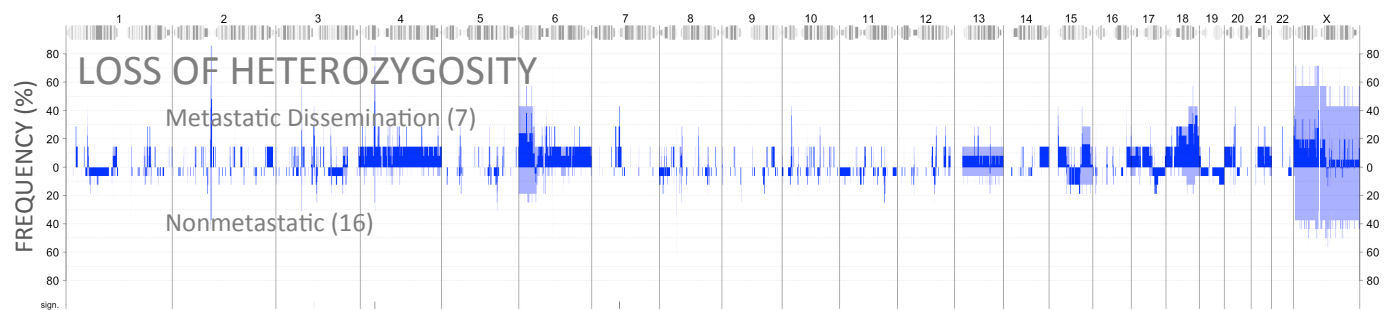

Supplement: Supplementary file 1 — Additional file 1: This file includes supplementary figures, genome-wide copy number estimates and statistics. (ZIP 5 MB) [file 12885_2014_5046_MOESM1_ESM.zip › Supplementary Data/Figure S1.pdf]

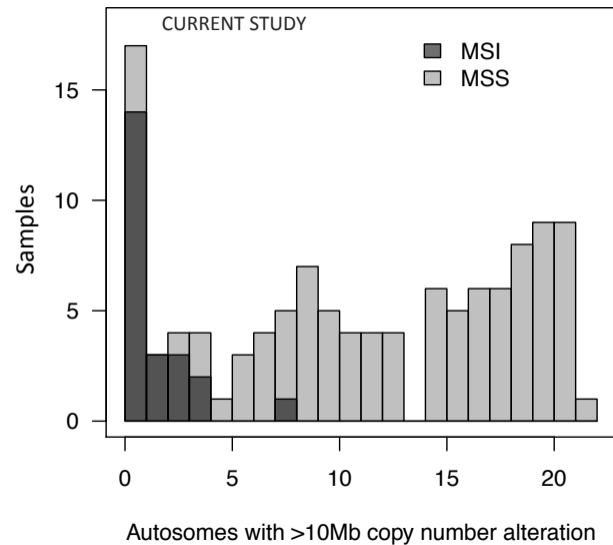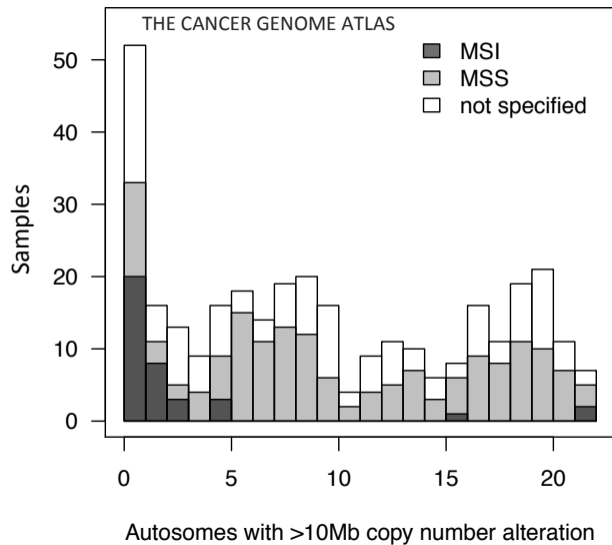

Supplement: Supplementary file 1 — Additional file 1: This file includes supplementary figures, genome-wide copy number estimates and statistics. (ZIP 5 MB) [file 12885_2014_5046_MOESM1_ESM.zip › Supplementary Data/Figure S2.pdf]

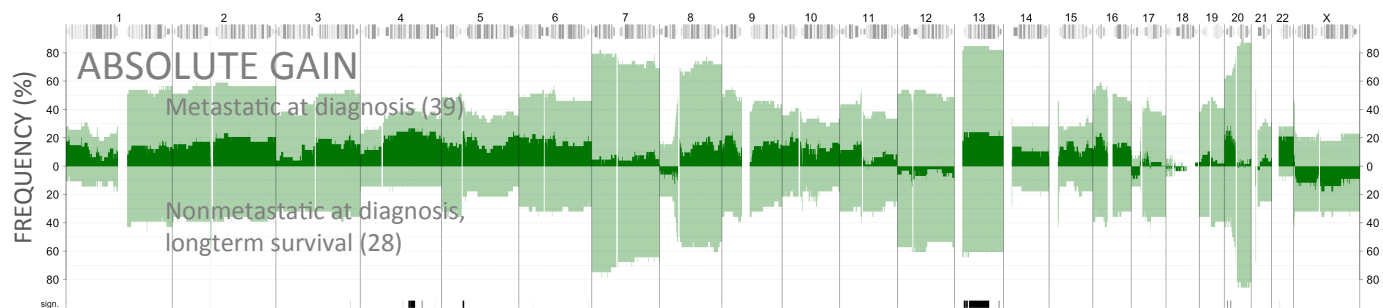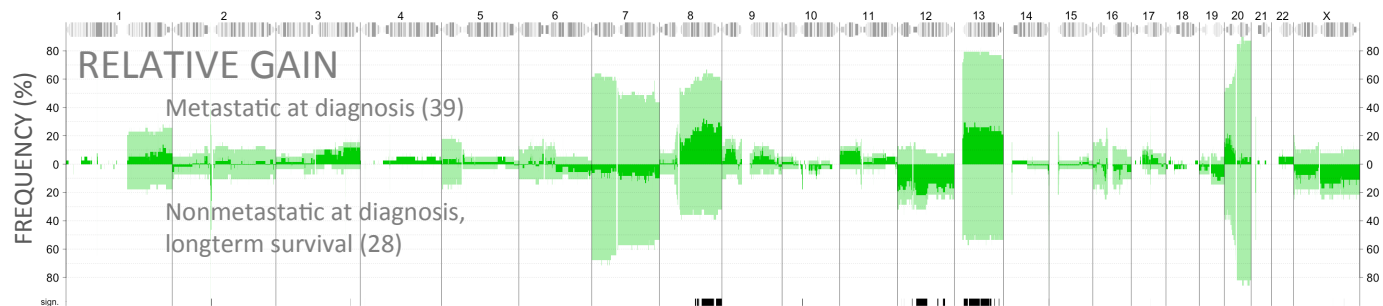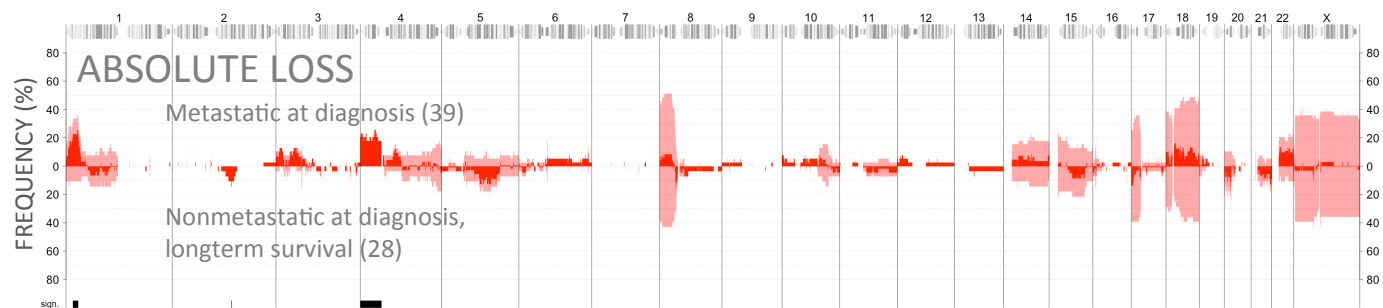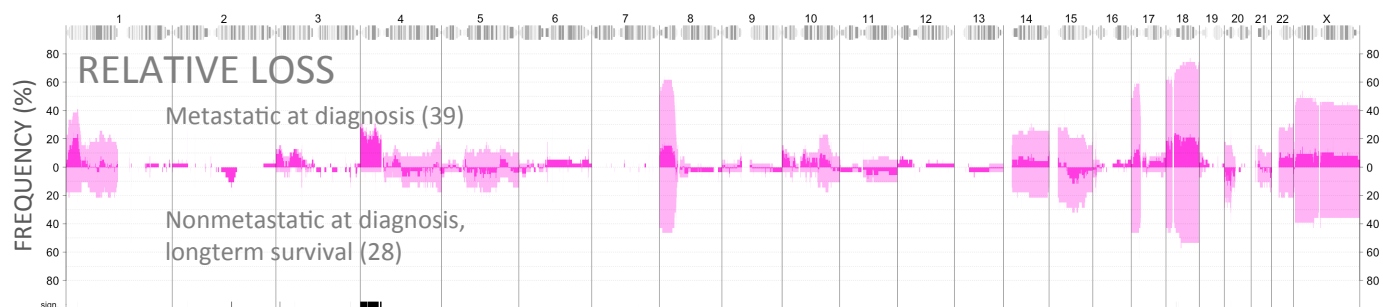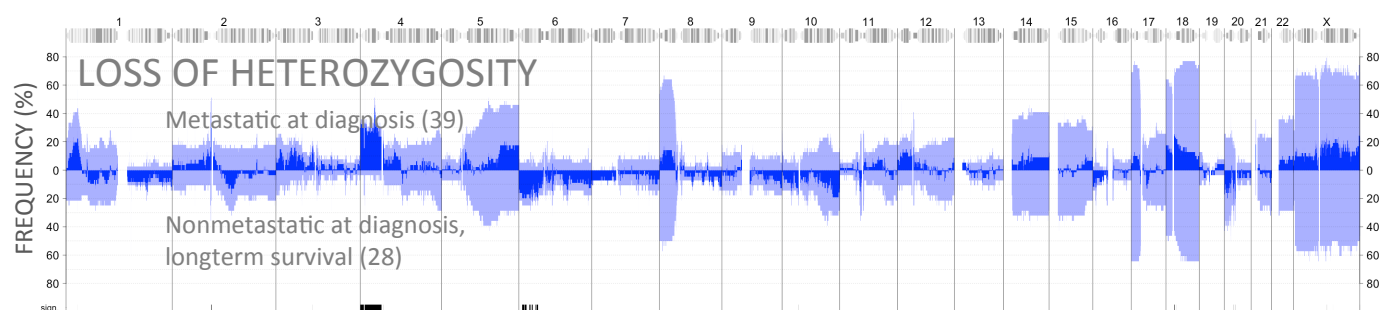

Supplement: Supplementary file 1 — Additional file 1: This file includes supplementary figures, genome-wide copy number estimates and statistics. (ZIP 5 MB) [file 12885_2014_5046_MOESM1_ESM.zip › Supplementary Data/Figure S3.pdf]
